# Clinical Impact of Cardiovascular Magnetic Resonance in Cancer Patients With Suspected Cardiomyopathy

**DOI:** 10.3389/fcvm.2021.734820

**Published:** 2021-10-26

**Authors:** Giv Heidari-Bateni, Jean-Bernard Durand, Cezar Iliescu, Greg Gladish, Anita Deswal, Amit R. Patel, Peter Kim, Juhee Song, Saamir Hassan, Nicolas Palaskas, Lauren A. Baldassarre, Chiara Bucciarelli-Ducci, Juan Lopez-Mattei

**Affiliations:** ^1^Department of Cardiology, Loma Linda University Medical Center, Loma Linda, CA, United States; ^2^Department of Cardiology, MD Anderson Cancer Center, Houston, TX, United States; ^3^Department of Thoracic Imaging, MD Anderson Cancer Center, Houston, TX, United States; ^4^Cardiology Division, Department of Medicine, University of Chicago, Chicago, IL, United States; ^5^Department of Biostatistics, The University of Texas MD Anderson Cancer Center, Houston, TX, United States; ^6^Cardiovascular Medicine, Yale School of Medicine, New Haven, CT, United States; ^7^Royal Brompton and Harefield Hospitals, Guy's and St Thomas' NHS Foundation Trust, King's College London, London, United Kingdom

**Keywords:** cardiovascular magnetic resonance, cardiomyopathy, Cardio-Oncology, clinical impact, echocardiography

## Abstract

**Objectives:** To assess the clinical impact of Cardiovascular Magnetic Resonance (CMR) in clinical decision making of cancer patients with a suspected cardiomyopathy in a tertiary cancer center.

**Background:** Cardiomyopathies of diverse etiologies are frequently encountered in a Cardio-Oncology practice. The clinical impact of CMR after a presumptive diagnosis of cardiomyopathy has not been studied in cancer patients.

**Methods:** We reviewed data on cancer patients with presumptive diagnosis of cardiomyopathy who underwent CMR in a tertiary cancer center. The clinical impact of CMR was defined as either change in clinical diagnosis or management post CMR results. Univariate and multivariate logistic regression models were used to assess whether any of the baseline characteristics were predictive of the clinical impact of CMR.

**Results:** A total of 110 consecutive patients were identified. Clinical impact of CMR was seen in 68 (62%) patients. Change in the clinical diagnosis and management was seen in 56 (51%) and 41 (37%) of patients, respectively. The most common change was prevention of endomyocardial biopsy in 26 patients (24%). Overall, patients with higher left ventricular ejection fraction (LVEF) by echocardiography (echo), clinical impact was influenced more by CMR (LVEF of 37.2 ± 12.3% vs. 51.5 ± 11.6%, *p* < 0.001). Cancer diagnosis of multiple myeloma was associated with change in the management post CMR (adjusted OR of 25.6, 95% CI 4.0–162.4, *p* = 0.001). Suspicion of infiltrative cardiomyopathy was associated with a higher likelihood of change in diagnosis. Having an LVEF≥40 by echo was associated with change in diagnosis and management by CMR.

**Conclusions:** Utilization of CMR has a significant clinical impact in cancer patients with suspected cardiomyopathy. Patients with cancer diagnosis of multiple myeloma, suspicion of infiltrative cardiomyopathy and those with higher LVEF by echo seem to benefit more from CMR.

## Introduction

The diagnosis and management of cardiomyopathies are important components of a Cardio-Oncology practice, given cancer patients are at an increased risk of heart failure (HF) (1) due to co-existing risk factors as well as cardiotoxic cancer therapeutics. The advancement of newer cancer therapies and improvement of survival rates, has led to an increase of the number of patients with cancer related cardiomyopathy (2, 3). In survivors of breast cancer, for instance, the adjusted 3-year cumulative incidence of anthracycline associated cardiomyopathy was calculated at 20.2 per 100 patients with an estimated increase by 21.7 per 100 patients with addition of trastuzumab (4).

Cardiovascular magnetic resonance (CMR) has been deemed appropriate for initial and sequential evaluation of patients with cardiomyopathies (specifically infiltrative, hypertrophic, and any cardiomyopathies of unclear etiology) based on appropriateness criteria from multimodality imaging scientific societies (5, 6). Moreover, CMR has an emergent role in detecting cardiotoxicity-related cardiomyopathy and other cardiovascular effects in patients undergoing anti-cancer therapy. Data from EuroCMR has shown that CMR has a strong impact on patient management, showing 62% of its findings impacting patient management (7). However, no data in cancer patients has been published in this regard.

In the current study, we aim to assess the clinical impact of CMR in clinical decision making for cancer patients with suspected cardiomyopathy in a large tertiary cancer center.

## Methods

**Figure 1:**
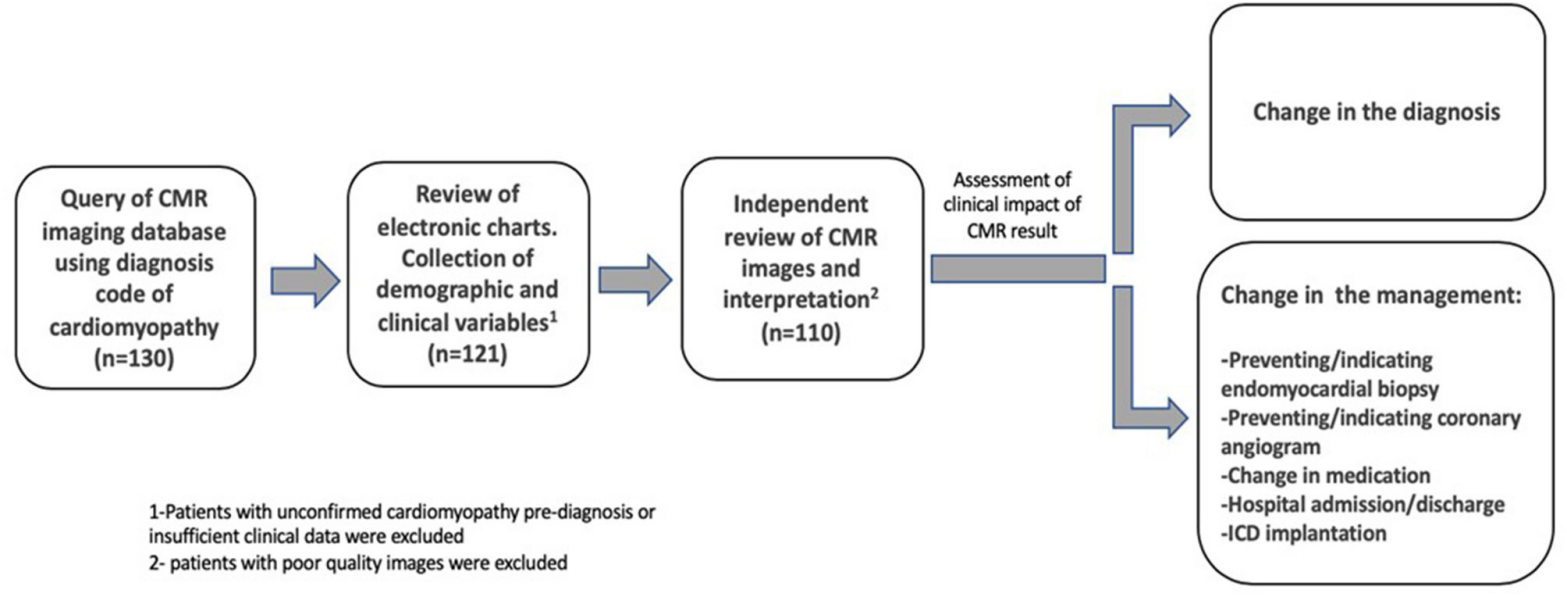
Algorithm of study design: cohort selection.

We designed a retrospective cohort study of patients treated at the MD Anderson Cancer Center in Houston, TX, United States. We queried a CMR imaging database from May 2015 to September 2017 to identify consecutive patients who underwent CMR for clinically suspected cardiomyopathy. All patients included were receiving cancer treatment at our institution. The study protocol was approved by the institutional review board of MD Anderson Cancer Center. We included 110 consecutive patients that underwent CMR in either inpatient or outpatient settings. The diagnosis of cardiomyopathy was pre-established clinically by chart review and available echocardiographic findings in all inpatient cases and in the majority of outpatient cases. For a minority of cases referred from outside centers, a recent outside hospital echocardiography record was used. Figure 1 illustrates a summary of study design algorithm. Given the diversity of cancer diagnosis in our cohort, we separated them into the following groups by cancer: solid tumors (most common was breast cancer, constituting 31% of the group), leukemia, lymphoma, myeloma and miscellaneous (more rare hematologic cancers).

All CMR images were acquired using a 1.5-T MRI scanner which was either Siemens Avanto (Siemens, Erlangen, Germany) or a 1.5-T GE AW (GE, Milwaukee, WI). All CMR exams were protocolled for cardiomyopathy, and included the following sequences: SSFP cines (real time cines if suspicion of constrictive pericarditis), T1 and T2 weighted double inversion recovery (IR) sequences, some included T2^*^, T1 (native and post contrast) and T2 mapping (Modified Look-Locker IR). Late gadolinium enhancement (LGE) was performed for tissue characterization using a segmented inversion-recovery sequence (in-plane spatial resolution, 1.8 × 1.3 mm; slice thickness, 8 mm; temporal resolution, 160–200 ms) 10–15 min after intravenous contrast administration (gadopentetate dimeglumine, 0.125 mmol/kg).

CMR images were interpreted independently by either a level 3 CMR board-certified cardiologist or a radiologist with level 3 equivalent training. Studies were reviewed to assess for image quality. Cases with poor quality studies, including those where gadolinium-based contrast was not administered, were excluded. For all patients, demographic and clinical variables including age, sex, type of malignancy, comorbidities, history of previous chemotherapy, and echocardiographic parameters were collected by electronic chart review. Patients with insufficient clinical data were excluded. The clinical impact of CMR was then assessed independently and determined upon consensus by two investigators (J.C.L., G.HB). We adopted the definitions per Abbasi et al. (8) where they defined significant clinical impact of CMR as either finding an entirely new diagnosis or if a change in clinical management occurred after CMR results. Change in diagnosis was defined as a diagnosis resulting from CMR that was previously unconfirmed or unsuspected. Change in management was defined as CMR results preventing or resulting in a procedure (invasive or medical), or admission or discharge from hospital (Figure 1). Changes in medical management that we considered as significant was either starting guideline directed medical treatment for heart failure or stopping it, starting or stopping anticoagulation, but no changes in cancer treatment were seen caused from CMR findings. Our outcomes were predefined as either clinical impact of CMR, depending on changes in diagnosis and changes in management. No survival analyses or hard endpoints were evaluated, just utilization endpoints as previously defined.

We evaluated patients' baseline clinical characteristics to assess if the clinical impact of CMR can be predicted by these characteristics. Univariate and multivariate logistic regression models were used to identify variables that were significantly associated with diagnosis change (change in diagnosis vs. no change), management change (change in management vs. no change), or either of them (any change vs. no change). Only variables with significant *p*-value in univariable analyses, or those with a trend toward a significant *p* value, were used in the multivariable analysis. Hosmer-Lemeshow test was used to check the model adequacy. A *p*-value less than 0.05 indicated a statistical significance. All statistical analyses were performed in SAS® Version 9.4 (SAS Institute, Cary, NC).

## Results

A total of 110 patients with clinical suspicion of cardiomyopathy were identified; of those 58 (53%) were female. The average age was 59 ± 15 years. Solid tumors were the most prevalent malignancies (40%), followed by myeloma (19%), and lymphoma (18%). Table 1 summarizes baseline demographic and clinical characteristics of the patients. Indications for CMR with respective percentage frequencies were the following: Routine CMR for cardiomyopathy (64%), suspected infiltrative cardiomyopathy (25%), hypertrophic cardiomyopathy (6%), viability (3%), suspected arrhythmogenic right ventricular dysplasia (2%) and carcinoid heart disease (1%) (see Table 2). Following CMR, cardiomyopathies were categorized into six different diagnostic groups as summarized in Table 3. In 27 (25%) patients with suspected iron overload cardiomyopathy, amyloidosis, or suspected myocarditis, CMR showed no evidence of cardiomyopathy (normal ejection fraction, normal T2^*^ and absence of late gadolinium enhancement) despite clinical suspicion and suggestive echocardiographic findings.

**Table 1:** Baseline demographic and clinical characteristics of the patients.

**Variable**	**Result^**a**^**
Age (years)^b^	59 ± 15
Sex	
Male	52 (47%)
Female	58 (53%)
Type of malignancy	
Solid tumors	45 (41%)
Leukemia	14 (13%)
Lymphoma	20 (18%)
Multiple Myeloma	21 (19%)
Miscellaneous	10 (9%)
Ejection fraction by echocardiography (%)^b^	42± 13
Ejection fraction <40%	32 (29%)
Diabetes	35 (32%)
Hypertension	65 (59%)
Atrial fibrillation	24 (22%)
History of chest radiotherapy	22 (21%)
History of coronary artery disease	27 (25%)
History of treatment with anthracycline	39 (36%)

a *Data are expressed as the number of cases (percentage of total) unless indicated otherwise*.

b *Data are expressed as the mean ± standard deviation*.

**Table 2:** CMR indications.

**Variable**	**Frequency (%)**
Routine CMR for cardiomyopathy	70 (64%)
Suspected infiltrative cardiomyopathy	27 (25%)
^*^Suspected hypertrophic cardiomyopathy	7 (6%)
^*^Viability	3 (3%)
^*^Suspected arrhythmogenic right ventricular dysplasia	2 (2%)
^*^Carcinoid heart disease	1 (1%)

* *For further analysis these categories were included in Other CM in Table 3*.

**Table 3:** Final CMR diagnosis.

**Diagnosis**	**Frequency^**a**^**
NICM	49 (45%)
ICM	8 (7%)
Cardiac amyloidosis	6 (5%)
HCM	6 (5%)
Other CM	14 (13%)
Non compaction	1 (1%)
Takotsubo	1 (1%)
Myocarditis	4 (4%)
Chagas	1 (1%)
Iron overload	1 (1%)
RV failure [PH]	3 (3%)
Constrictive pericarditis	1 (1%)
Eosinophilic CM	1 (1%)
No cardiomyopathy	27 (25%)

a *Data are expressed as the number of cases (percentage of total). CMR, cardiac magnetic resonance imaging; NICM, non ischemic cardiomyopathy; ICM, Ischemic cardiomyopathy, HCM, hypertrophic cardiomyopathy; CM, cardiomyopathy; RV, right ventricle; PH, pulmonary hypertension*.

Overall, the clinical impact of CMR was seen in 68 (62%) patients. Results of CMR changed the diagnosis in 56 (51%), the management in 41 (37%), and both management and diagnosis in 29 (26%) patients. The most common clinical impact of CMR was prevention of endomyocardial biopsy in 26 (24%) patients by ruling out the working diagnosis of suspected infiltrative cardiomyopathy. One noticeable finding was that in 42 patients (38%) there was no change in diagnosis or management, and the mean LVEF in this group by TTE was 37 ± 12% in contrast to the 29 patients that had changes in both diagnosis and management, whose corresponding mean LVEF by TTE was 51 ± 11% (*p* < 0.001 by Chi-square test).

**Figure 2:**
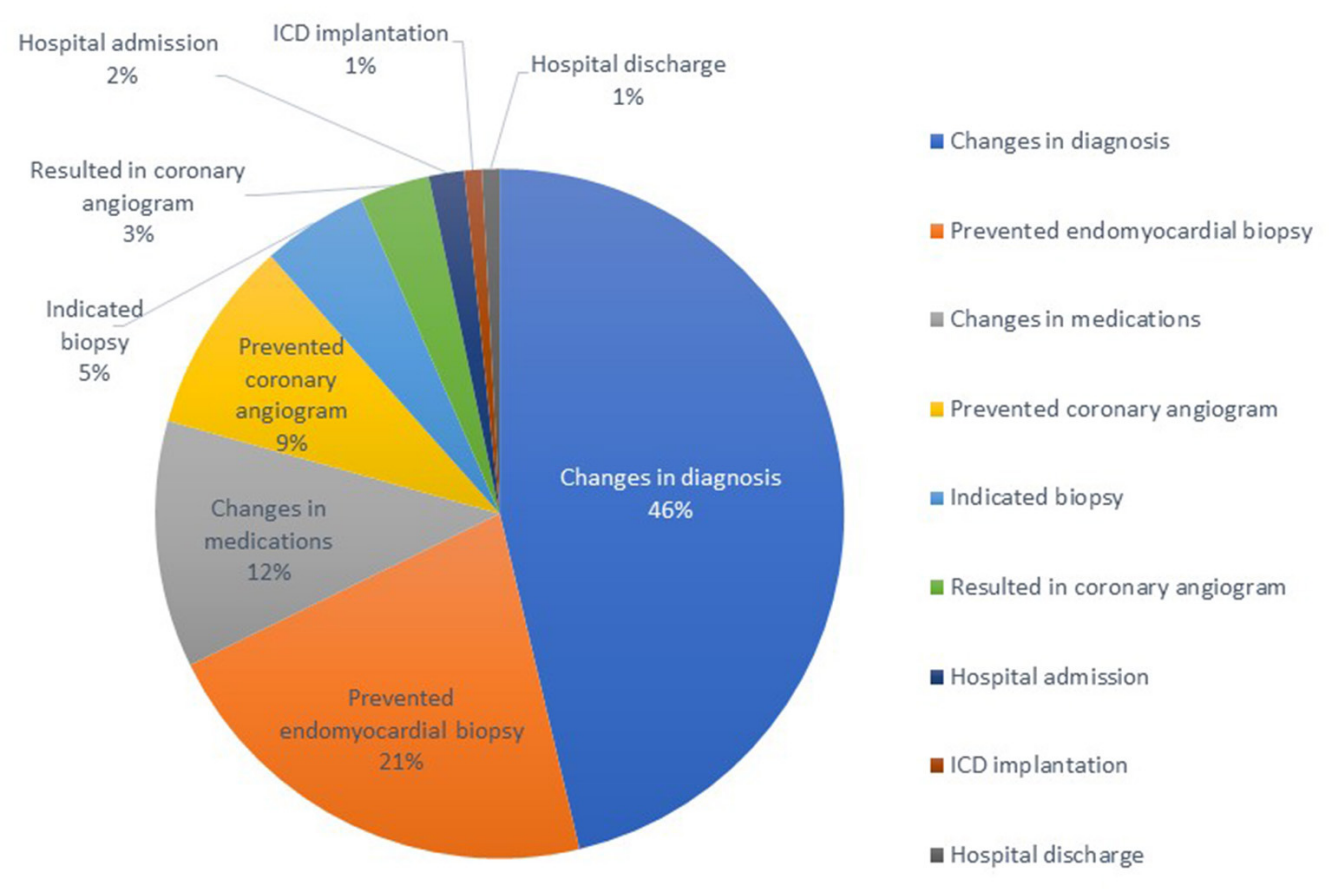
Clinical impact of utilization of cardiac magnetic resonance in a tertiary cancer center: 121 events in total of 66 patients.

Table 4 summarizes all the management changes post CMR. Examples of patients with change in both diagnosis and management are summarized in Table 5. The clinical impact of CMR is illustrated in Figure 2. To assess whether any of the baseline characteristics can predict the clinical impact of CMR, univariate and multivariate logistic regression models were fitted, and they are summarized in Tables 6, 7. All analyses demonstrated that a higher ejection fraction by echocardiography (LVEF ≥ 40) predicts a greater clinical impact of CMR, adjusting for type of malignancy (adjusted OR 7.09, 95% CI, 2.09–24.11, *p* value = 0.002 and 6.16 with 95% CI 1.47–25.77, *p* value = 0.013 for change in diagnosis and management respectively). For change in diagnosis, a suspicion of infiltrative cardiomyopathy, when compared with routine indication of CMR for cardiomyopathy had a higher likelihood of change in diagnosis in the multivariate analysis for change in diagnosis (adjusted OR: 10.03, 95% CI, 1.91–52.69, *p* value = 0.006), but not in multivariate analysis for change in management.

**Table 4:** Summary of change in management followed by CMR results (*n* = 110).

**Change in management**	**Frequency^**a**^**
Prevented endomyocardial biopsy	26 (24%)
Change in medications	14 (13%)
Prevented coronary angiogram or PCI	11 (10%)
Resulted in endomyocardial biopsy	6 (5%)
Resulted in LHC	4 (4%)
Hospital admission	2 (2%)
Hospital discharge	1 (1%)
Resulted in ICD implantation	1 (1%)

a *Data are expressed as the number of cases (percentage of total). Some patients had change in more than one management category. CMR, cardiac magnetic resonance imaging; PCI, percutaneous coronary intervention; LHC, left heart catheterization; ICD, implantable cardioverter-defibrillator*.

**Table 5:** Examples of patients with (green) and without (red) changes in both diagnosis and management after CMR.

**Patient description**	**Indication for CMR**	**CMR findings**	**Clinical impact**
68 y/o F with infiltrative ductal carcinoma, PMHx of HLP and HTN. LVEF of 40-45% in echo with poor acoustic windows	Work up on etiology of cardiomyopathy and better assessment of EF prior to next round of chemotherapy	Inferior wall subendocardial infarction with partial viability in the RCA territory	Incidental finding of ischemic cardiomyopathy; underwent left heart catheterization
66 y/o M with Smoldering multiple myeloma, PMHx of diabetes. Echo finding of LVH and diastolic dysfunction.	Suspicion of infiltrative cardiomyopathy based on the echocardiogram findings	Hypertrophic cardiomyopathy; cardiac amyloidosis ruled out	Hypertrophic cardiomyopathy: no endomyocardial biopsy pursued
57 y/o with HTN, CLL and bladder cancer that had an LVEF of 40-45% by Echo	Work up on etiology of cardiomyopathy	Mid myocardial hyperenhancement in basal septum. LVEF: 52%. Diagnosis of NICM	No change in management as GDMT for HF was started with echo results. No change in diagnosis as echo findings and clinical presentation suggested NICM
62 y/o F with breast cancer and prior treatment with anthracycline, her Echo showed an LVEF of 39%	Work up on etiology of cardiomyopathy	Findings suggestive of non-ischemic cardiomyopathy. CMR LVEF of 37%.	No change in management as GDMT for HF was started with echo results. Echo identified correctly the clinical diagnosis.

*CMR, cardiac magnetic resonance imaging; y/o, years old; F, female; M, male; PMHx, past medical history; HLP, hyperlipidemia; HTN, hypertension; EF, ejection fraction; RCA, right coronary artery; MGUS, monoclonal gamopathy of unknown significance; LV, left ventricle; NICM, non-ischemic cardiomyopathy; GDMT, guideline directed medical therapy; HF, Heart Failure*.

**Table 6:** Selected univariate and multivariate analysis of factors predicting diagnosis change following CMR.

**Variable**	**Univariate logistic regression model**			**Multivariate logistic regression model** ^**c**^		
	**OR**	**95% CI**	***P*-value**	**aOR**	**95% CI**	***P*-value**
Age	1.03	0.99–1.05	0.079	1.05	1.005–1.09	0.031
Female sex	0.77	0.35–1.72	0.521			
Type of malignancy^a^						
Lymphoma	0.73	0.22–2.37	0.596			
Leukemia	1.94	0.54–6.91	0.308			
MM	9.68	1.97–47.52	0.005			
Miscellaneous	4.24	0.79–22.84	0.093			
CMR indication^b^						
Infiltrative CM	12.29	2.66–56.83	0.001	10.03	1.91–52.69	0.006
Other	0.48	0.13–1.71	0.255	0.31	0.06–1.54	0.153
Diabetes	2.56	1.03–6.34	0.043			
Hypertension	1.05	0.46–2.38	0.905			
Atrial fibrillation	3.41	1.15–10.15	0.028			
Echocardiographic EF	1.08	1.04–1.13	<0.001			
EF≥40	5.35	1.99–14.38	0.001	7.09	2.09–24.11	0.002
EF≥50	7.21	2.60–19.98	<0.001			
History of treatment with anthracycline	0.33	0.14–0.80	0.014			

*CMR, cardiac magnetic resonance imaging; OR, Odds Ratio; CI, confidence interval; aOR, adjusted odds ratio; EF, ejection fraction; N/A, not applicable (variable not included in the multivariate logistic regression model); CM, cardiomyopathy*.

a *“Solid tumor” malignancy as reference group*.

b *“Routine CMR for cardiomyopathy” as reference group*.

c *The multivariate model initiated with the following variables: age, type of malignancy, diabetes, atrial fibrillation, EF, CMR indication, and history of anthracycline use. It reduced by stepwise selection to age, CMR indication, and EF as shown here. Same results obtained when using of EF ≥ 50% in lieu of ≥ 40% (adjusted OR of 7.57 with 95% CI 1.42–40.24, p value = 0.018 and adjusted OR of 9.13 with 95% CI 2.23–37.45, p value = 0.002 for CM infiltrative and EF≥50, respectively)*.

**Table 7:** Selected univariate and multivariate analysis of factors predicting management change following CMR.

**Variable**	**Univariate logistic regression model**			**Multivariate logistic regression model** ^**c**^		
	**OR**	**95 % CI**	***P*-value**	**aOR**	**95% CI**	***P*-value**
Age	1.01	0.98–1.04	0.443			
Female sex	1.43	0.59– 3.45	0.424			
Type of malignancy^a^						
Lymphoma	1.61	0.48–5.44	0.444	0.86	0.18–4.04	0.845
Leukemia	1.84	0.41–8.33	0.429	1.25	0.23–6.73	0.799
MM	18.40	3.55–95.50	0.001	25.56	4.02–162.44	0.001
Miscellaneous	4.60	0.72–29.33	0.106	3.99	0.33–48.36	0.277
CMR indication^b^						
Infiltrative CM	12.68	2.67–60.33	0.001			
Other	0.16	0.02–1.33	0.089			
Diabetes	1.90	0.71–5.06	0.199			
Hypertension	1.18	0.49–2.86	0.716			
Atrial fibrillation	2.03	0.62–6.67	0.246			
Echocardiographic EF	1.07	1.03–1.04	0.001			
EF≥40	4.54	1.60–12.86	0.004	6.16	1.47–25.77	0.013
EF≥50	5.01	1.73–14.51	0.003			
History anthracycline use	0.44	0.18–1.11	0.084			

*CMR, cardiac magnetic resonance imaging; OR, Odds Ratio; CI, confidence interval; aOR, adjusted odds ratio; EF, ejection fraction; N/A, not applicable (variable not included in the multivariate logistic regression model); CM, cardiomyopathy*.

a *“Solid tumor” malignancy as reference group*.

b *“Routine CMR for cardiomyopathy” as reference group*.

c *The multivariate model initiated with the following variables: type of malignancy, CMR indication, diabetes, EF, and history of anthracycline use. It reduced by stepwise selection to type of malignancy and EF as shown here. Same results obtained when using of EF ≥ 50% in lieu of ≥40% (adjusted OR of 16.92 with 95% CI 2.94–97.38, p value = 0.002 and adjusted OR of 3.85 with 95% CI 1.11–13.34, p value = 0.034 for MM and EF ≥ 50, respectively)*.

We also found that “type of malignancy” predicted change in management post CMR. As shown in Table 7, multiple myeloma was the cancer group associated with significant change in the management post CMR (adjusted OR of 25.56 with 95% CI 4.02–162.44, *p* value = 0.001).

## Discussion

This study demonstrates a valuable role for CMR as part of the assessment for suspected or known cardiomyopathy in patients with cancer. In 62% of our patients there was a benefit from the addition of CMR imaging in their diagnostic work up, by achieving either a change in diagnosis or change in management. Furthermore, we identified baseline clinical characteristics that could predict the clinical impact of CMR. In particular, we showed that patients with higher left ventricular ejection fraction by echocardiography, patients with a diagnosis of multiple myeloma as the primary malignancy and those with suspicion of infiltrative cardiomyopathy were those in which CMR had the most clinical impact.

CMR has proven to have an additive role in diagnosis and management of patients with cardiomyopathy (8–10). However, in patients with non-ischemic cardiomyopathy, its value on routine use may be limited, given it may not yield more specific etiologies in majority of cases (11). Which was consistent with our findings, given routine CMR for cardiomyopathy didn't yield as much clinical impact as suspicion of infiltrative cardiomyopathy. Compared to echocardiography, CMR has a higher spatial resolution, larger field of view, highly reproducible ventricular volumes and ejection fraction quantification (12) with the ability for functional assessment and ability to perform tissue phenotyping using tissue characterization sequences such T1 weighted imaging with late gadolinium enhancement (LGE) (13), T2 weighted imaging and parametric mapping (14).

With the advancement of CMR techniques, recent multi-society expert consensus recommendations for multimodality imaging in cardiac amyloidosis have considered a central role for CMR in the non-invasive diagnosis of cardiac amyloidosis (15). CMR is a cornerstone test in the evaluation of patients with left ventricular hypertrophy (LVH) phenotype on echocardiography and suspected infiltrative cardiomyopathies, explaining why there was a significant clinical impact in cancer patients with suspected infiltrative cardiomyopathy particularly those with primary multiple myeloma, given the risk to develop cardiac amyloidosis. This might in part explain our findings of baseline multiple myeloma predicting the clinical impact of CMR.

Multivariate analyses also revealed that the clinical impact of CMR in both diagnosis and management is more appreciated in patients' groups with an echo LVEF of 40% or higher and changes in diagnosis more likely with suspicion of infiltrative cardiomyopathy (adjusted OR 10.03, *p* = 0.006). This could be explained due to the high proportion of cases (25%) that CMR showed no evidence of cardiomyopathy despite clinical suspicion by TTE. This finding changes the management dramatically, as patients could be able to resume chemotherapy promptly and without the need for treatment of Heart failure. Conversely, the patients that did not have significant change in diagnosis and management had lower mean LVEF by TTE, which could suggest that in most of these cases LV systolic dysfunction was identified appropriately by TTE. In other words, in the context of a significant decrease in LVEF that could be detected by TTE, CMR might not add to the diagnosis or management. Anthracycline and trastuzumab cardiotoxicity and related cardiomyopathies are not well characterized in CMR by a specific patterns of LGE or specific findings in parametric mapping (16). Conversely, if an LVEF measurement by TTE is borderline low or in the realm of 40%, CMR accurately differentiates true decreases from cases of preserved LV systolic function because of its robustness in reproducibility and less interobserver variability in ventricular volumes and LVEF quantification. This suggest that routine utilization of CMR for cardiomyopathy evaluation based on echo findings might not offer clinical benefit if echo identifies well possible etiologies such as decreased LVEF from anthracyclines. Also suspicion of infiltrative cardiomyopathy was associated with increased of change in diagnosis related with CMR. Which suggests that clinical impact by CMR is higher in patients for which iron overload cardiomyopathy or cardiac amyloidosis must be evaluated. CMR ruled out cardiomyopathy in 25% of patients. This has an impact for patients' mental wellbeing, withdrawal of heart failure medications and resumption of lifesaving cancer therapies. In our study, prevention of endomyocardial biopsy was found to be the most common clinical impact of CMR comprising 24% of patients. Prevention of endomyocardial biopsy is important in the setting of cancer particularly for those actively receiving chemotherapy given the increased risk of complications, mainly vascular complications and bleeding in this vulnerable group. A cost effectiveness analysis may also enlighten the economic benefit of using CMR as a gatekeeper for myocardial biopsy.

In conclusion, application of CMR in Cardio-Oncology appears to have frequent clinical impact (62% patients) on the evaluation of confirmed or suspected cases of cardiomyopathy in a cohort of cancer patients. Baseline systolic function from TTE, suspicion of infiltrative cardiomyopathy and primary malignancy type increase the likelihood of clinical impact of the addition of CMR to the diagnostic approach. Our findings support an important role of CMR in a Cardio-Oncology practice. Further larger and multi-center studies looking at hard clinical endpoints and cost-effectiveness analyses are needed to quantify better the benefits of CMR in these patients.

## Limitations

We cannot exclude the role of selection and referral bias of the primary cardiologist when choosing the appropriate patient for CMR assessment; however, the studied patients represents a heterogeneous group either referred by outpatient centers or seen as an inpatient consult in a tertiary cardio-oncology practice.

Our study has some additional limitations. First, this is a retrospective study which can be skewed by limitations of medical documentation and the absence of a control group. Second, there are some known limitations in application of CMR such as patients with claustrophobia, prosthetic devices or foreign bodies. Also, 11 out of 121 (9%) of scans were excluded due to poor image quality or lack of contrast (see Figure 1), and therefore our results may overestimate the benefit of performing CMR.

Moreover, the economic value of CMR for all patients with suspected cardiomyopathy is uncertain. Similarly, there is no outcome data on survival benefit of using CMR in the management of cardiomyopathy in cancer patients. Undoubtedly a cost-effective analytical study or a comparative effectiveness study with focus on survival benefit can better highlight the value of this approach.

### Clinical Perspectives

In patients with cancer and suspected cardiomyopathy, CMR may result in change in diagnosis and management in certain clinical scenarios. Patients with LVEF of 40% or more, those with suspicion of infiltrative cardiomyopathy and those with cancer diagnosis of multiple myeloma, have a higher likelihood to benefit from the use of CMR.

Further prospective research is needed to identify the value, including the economic burden, of the use of CMR for cancer patients with suspected cardiomyopathy.

## Data Availability Statement

The raw data supporting the conclusions of this article will be made available by the authors, without undue reservation.

## Ethics Statement

The studies involving human participants were reviewed and approved by MD Anderson IRB provided exemption due to retrospective review. Written informed consent for participation was not required for this study in accordance with the national legislation and the institutional requirements.

## Author Contributions

GH-B and JL-M planned and wrote the manuscript. All other authors edited the manuscript.

## Funding

The statistical analysis work was supported in part by the Cancer Center Support Grant (NCI Grant P30 CA016672). CB-D was supported by the NIHR Biomedical Research Centre at University Hospitals Bristol NHS Foundation Trust and the University of Bristol.

## Author Disclaimer

The views expressed in this publication are those of the author(s) and not necessarily those of the NHS, the National Institute for Health Research or the Department of Health and Social Care.

## Conflict of Interest

The authors declare that the research was conducted in the absence of any commercial or financial relationships that could be construed as a potential conflict of interest.

## Publisher's Note

All claims expressed in this article are solely those of the authors and do not necessarily represent those of their affiliated organizations, or those of the publisher, the editors and the reviewers. Any product that may be evaluated in this article, or claim that may be made by its manufacturer, is not guaranteed or endorsed by the publisher.

## Abbreviations

CMR: Cardiovascular Magnetic Resonance
TTE: Transthoracic echocardiogram
LVEF: Left ventricular ejection fraction
LGE: Late Gadolinium enhancement.
